# The Management and Implementation of Online Dementia Caregiver Support Groups: A Proposed Framework for Guidelines

**DOI:** 10.1002/alz70858_106009

**Published:** 2025-12-25

**Authors:** Amy M Almeida, Katherine Brandt, Brad C. Dickerson

**Affiliations:** ^1^ MGH Frontotemporal Disorders Unit, Boston, MA, USA; ^2^ Frontotemporal Disorders Unit, Charlestown, MA, USA; ^3^ Frontotemporal Disorders Unit and Massachusetts Alzheimer's Disease Research Center, Department of Neurology, Massachusetts General Hospital and Harvard Medical School, Boston, MA, USA

## Abstract

**Background:**

Caregivers of individuals living with dementia are at increased risk for social isolation, stress‐related illnesses, and other adverse outcomes. Participation in a caregiver support group can provide connections to others in similar situations and to resources and strategies, all of which likely reduce adverse outcomes. Prior to the COVID‐19 pandemic, caregiver support groups were primarily conducted in‐person. With the proliferation of online support groups post‐pandemic, we believe a framework for optimal online support group management and implementation is needed to prioritize caregiver safety and well‐being.

**Method:**

Due to the COVID‐19 pandemic, the Boston‐area frontotemporal dementia (FTD) caregiver peer support group shifted to meeting weekly via Zoom in March 2020.

Trained support group facilitators (SGFs) attended every meeting to manage online meeting technology, promote adherence to group guidelines, and record meeting notes.

New members were screened to confirm FTD caregiver status and approved for registration. Once registered, they were added to the group mailing list and received weekly emails with meeting links and follow‐up resources.

**Result:**

Since March 2020, 282 unique caregivers have registered for support group and 228 have attended at least one session. Attendance each year has increased from 426 group attendees in to 914 in 2024. Average weekly attendance has grown from 11 members per meeting in 2020 to 18 in 2024. Support group members attended from fourteen states in 2024, including individuals from more rural areas without local in‐person support groups.

Barriers to attending support group have diminished due to online access. Support group members have provided qualitative feedback about areas of support group that were most valuable to them, including receiving weekly follow‐up emails, ease of technology, and sense of community with other members.

**Conclusion:**

The framework and guidelines developed by the SGFs for this support group create a safe and welcoming environment for caregivers to share all aspects of their caregiving journey. Providing caregivers of PLWD with a regular online support group with established guidelines for behavior can diminish the isolation that dementia caregivers often experience, promoting opportunity for high quality care for care recipients.

